# Adrenalin in Cardiac Toxemia of Pneumonia

**Published:** 1904-02

**Authors:** 


### Adrenalin in Cardiac Toxemia of Pneumonia.

HENRY L. ELSNER, Syracuse, N. Y., (*New York Medical Journal,* January 2, 1904,) directs attention to the appalling mortality of pneumonia due to the resulting cardiac toxemia. The prime factor in this disease is a toxemia with obstruction in the pulmonary circuit, leading to cardiac asthenia. Marked changes occur in the right half of the heart, with farreaching degenerative changes in the muscle, heart-clots, and vasomotor paralysis.

Three remedies meet the indications presented by the circulatory changes due to paralysis of the vasomotor centers, the dilated condition of the arteries and the weakened heart. These are strychnine, digitalis, and suprarenal extract, or adrenalin, its active principle. Adrenalin acts on the heart and bloodvessels favorably; it does not act on the vasomotor center. Hence, it may be used to assist strychnine. When the vasomotor center is exhausted and blood-pressure study proves the inefficiency of strychnine, adrenalin may still be administered, and, in some cases which seem unpromising, when combined with the method of stimulation about to be suggested, the patient may be carried beyond the critical period to a safe recovery. Suprarenal extract, or adrenalin, has seemed to the author to act as a needed food in all infections where there is danger of myocardial degeneration. He reports a case of pneumonia, in a woman, the mother of five children, in whom it had been impossible to raise a continually lowering blood pressure with strychnine. The systolic blood pressure was almost immediately raised by the repeated administration at short intervals of fifteen minims of a 1 to 1,000 solution of adrenalin hypodermatically, and the patient recovered.

